# When the times get tough the toughs get funny: Means by which humor buffers against death anxiety emerged during COVID-19 outbreak

**DOI:** 10.1371/journal.pone.0273338

**Published:** 2022-08-19

**Authors:** Michal Mahat-Shamir, Maya Kagan

**Affiliations:** School of Social Work, Ariel University, Ariel, Israel; La Sapienza University of Rome, ITALY

## Abstract

According to Terror Management Theory (TMT), there are three common buffers that minimize the anxiety of mortality salience: affirmation of a) one’s cultural worldview, b) the self and one’s personal values, and c) one’s significance in the context of close personal relationships. The current study aimed at examining the contents of memes, which were distributed on social media during the COVID-19 outbreak, to explore the means by which humor buffers against death anxiety. A deductive and inductive thematic analysis captured three means by which humor buffers against death anxiety, a) humor as a means for connecting to cultural worldviews; b) humor as a means for inclusion in group; c) humor as a means to gain a sense of control. These findings are discussed through the theoretical lens of TMT.

## Introduction

In December 2019, Wuhan and gradually other places in China experienced an outbreak of pneumonia epidemic caused by the 2019 novel coronavirus (COVID-19) [1]. The spread of COVID-19 has taken on pandemic proportions, affecting over 100 countries in a matter of weeks, including Israel [2, 3]. Thus, the World Health Organization (WHO) has declared the COVID-19 a public health emergency of international concern [4]. Keeping mortality as low as possible became the highest priority for individuals and governments; hence and in accordance with the WHO advice regarding measures for containing the epidemic, governments, including the Israeli government, implemented quarantine, social distancing, and isolation of infected populations [4].

Research has shown that health settings [5–8] and a threat to one’s close relationships [9, 10] increase the accessibility of death-related thoughts and death anxiety. Thus, it is understandable how these conditions, a life-threatening pandemic outbreak, and social distancing, may increase individuals’ death anxiety. In this regard, one of the most influential theories which explore the manner by which individuals deal with exposure to death-related stimuli, such as a life-threatening pandemic outbreak and social distancing, is the Terror management theory (TMT) [11] and its extension: anxiety buffer disruption theory [12].

### Terror management theory

One of the main theoretical postulations of TMT is that as humans we are aware of our mortality; this knowledge creates extreme levels of stress and anxiety. The inevitability of death and our perceived lack of control over its circumstances create an internal sense of anxiety, which makes life extremely difficult [11]. In other words, the juxtaposition of death awareness, presumably a uniquely human capacity made possible by cognitive abilities such as self-awareness and abstract thought, and the instinct for self-preservation, common to all animals, creates a challenging and harsh living situation [2, 13].

TMT holds that when faced with the reality of their own mortality, individuals cope with the anxiety (or ‘terror’) of this by drawing on sources of meaning that connect them to aspects of their selves or lives that will live on after they die [14]. The awareness of one’s mortality is known as the mortality salience effect. Greenberg et al. developed this theory into a testable psychological model in which existential terror is managed by an anxiety buffer [15]. The anxiety buffer has two components: the first is the belief in a cultural worldview; and the second is self-esteem, achieved when one feels he/she is living up to the standards set out by one’s cultural worldview. Pyszczynski and Kesebir extended the theory with their model of a tripartite terror-management system, comprising dynamically interrelated attachment, worldview, and self-esteem processes [12]. This model integrates attachment theory and terror management theory, proposing that much of human behavior is aimed at maintaining a sense of psychological security and reducing conscious and unconscious anxiety about personal vulnerability (and ultimately, death). Close relationships, cultural belief systems, and self-esteem all enhance psychological security and decrease death anxiety.

A threat to one’s health and death anxiety is not only logically related (i.e., illness may lead to death), but also appear to be driven by core, underlying emotion regulation mechanisms [2]. According to the two-factor model of death anxiety [16], this construct is determined by one’s life experiences regarding the topic of death as well as by overall health [17, 18]. In addition, social distancing and quarantine may also lead individuals to experience social isolation, an objective condition in which an individual has little contact with friends, family, or community [19, 20]. Social isolation and death are related constructs as they are members of a broader category of “emergency situations that can be remedied through coalitional support” (p. 373) [21]. Thus, as life-threatening pandemic outbreaks and social distancing create a mortality salience effect, individuals need to enhance their use of buffer mechanisms that minimize the anxiety of mortality salience. One way to utilize these psychological buffers is humor [22].

### Humor as an anxiety buffer

Research indicates that a sense of humor might reduce some anxieties level, provide positive effects on mental health, and is adaptive to cope with stressful situations [23–25]. Moreover, research shows that any positive or educating type of humor may reduce anxiety [26, 27].

Much of the theoretical and empirical psychology literature on humor takes a motivational approach [28]. A recurring theme in the motivational literature is humor’s function as a buffer against death anxiety caused by the awareness of our own mortality [22]. An individual who holds positive thinking and emotion may behave more positively and adaptively in the face of any stress, including death [29]. Existential perspectives on humor propose that humor’s primary function is to address mortality concerns; whilst we cannot control mortality’s inevitability, we can choose how we react to humorous material, which offers us a means of reasserting control [22, 30]. Other motivational accounts see humor as a means of facilitating social interactions [31]. Evolutionary theories of humor, emphasize the social function of jokes and laughter for enhancing group cohesion, either by strengthening bonds with members of one’s in-group, or by excluding or derogating members of one’s out-group [22, 32–36].

During the COVID-19 outbreak, we have noticed a tremendous number of memes, concerning the COVID-19 outbreak and the implication of the outbreak, spreading on social media. As Davison describes it: “An internet meme is a piece of culture, typically a joke which gains influence through online transmission” (p. 122) [37]. We believe this phenomenon reflects individuals’ attempt to utilize buffer mechanisms that minimize the anxiety of mortality salience effect created by the life-threatening pandemic and social distancing. In the current study, we seek to examine the contents of memes, which were distributed on social media, to explore the means by which humor buffers against death anxiety.

## Method

This research is a qualitative investigation based on memes, which were distributed on social media, as a means of data collection and thematic procedures as means of data analysis. Two forms of thematic analysis, both inductive and deductive, were implemented in this study. Thematic analysis is a search for themes that are deductively/inductively recognized/emerge and are perceived as being important to the description of the phenomenon [38].

### Data source

The data source consisted of memes distributed on WhatsApp and Facebook during February, March, and April 2020, the time of the COVID -19 outbreak in Israel. Memes can range from only a title or a line to 4 lines of text. On average the text of a meme consists of 1–3 lines. The memes collected are in Hebrew. Importantly, all of the memes were distributed openly and without restrictions on social media, thus, the collection and analysis method complies with the terms and conditions for the sources of the data.

### Participants

As the distribution of memes is often viral, there is no way to know who originated them. Thus, no formal demographic characteristics could be obtained regarding the individuals distributing the memes.

For data collection we used convenience sampling [39], a sampling method frequently used in both qualitative and quantitative studies [40] as indeed was used in a previous study that explored how the use of humor in memes served as a relief therapy in the face of COVID-19 [41].

In the first step of data collection, memes were collected by the researchers through their social media platforms. The second step consisted of asking friends and family members (from different generations: friends our age, our parents and their friends, and our teenage children and their friends) to share memes distributed to their social media platforms. These two steps led to 237 memes, distributed in Israel, concerning the COVID -19 outbreak. The final collection of memes was in line with Davison’s definition of memes: “An internet meme is a piece of culture, typically a joke which gains influence through online transmission” (p. 122) [37]. These memes included text only, with no images accompanying them. The criterion for selecting the memes was in line with Xu’s assertion that: “Humor that employs a combination of different contextual dimensions increases the value of the intended effects” (p. 117) [42]. Thus, the criterion for selecting the memes was the commonality of the theme i.e. humorous presentation of the Israeli life during the lockdown.

### Data analysis

The method of analysis chosen for this study was a hybrid approach of qualitative methods of thematic analysis, incorporating both the data-driven inductive approach of Boyatzis and the deductive, a priori template-of-codes approach outlined by Crabtree and Miller [43, 44]. These two forms of data analysis were perceived most relevant due to the juxtaposition of knowledge concerning death anxiety buffers and the way humor may act as a buffer against death anxiety found in the literature, and the dearth of scientific examination of this knowledge.

Deductive thematic analysis began with explanatory assumptions and/or provisional definitions of something to be explained [45]. Per the protocol of such an approach, the hypothesis or definition was then compared with the data and when they did not match, modifications were made. The inductive thematic analysis method was used in the modification process. Inductive thematic analysis refers to simultaneous comparing of all incidents observed for all memes and then arriving at a category label [45].

#### The analysis process

The deductive thematic analysis method was first used where the three common buffers that minimize the anxiety of mortality salience (affirmation of (a) one’s cultural worldview, (b) the self and one’s personal values, and (c) one’s significance in the context of close personal relationships) were found in the data and the metaphorical labels originate in the literature were checked to see if they accurately matched the words of the memes. Secondly, the method was used in its inductive way: each category of three common buffers that minimize the anxiety of mortality salience was examined to research an understanding of the manner these memes use this death anxiety buffer. Last, inductive thematic analysis was implemented to recognize ideas presented in the memes that did not match the three recognized common buffers that minimize the anxiety of mortality salience. Both researchers independently identified the same overall ideas and then came to an agreement on the final identification, categorization, and later modification. The final results included the main commonly used means by which humor buffers against death anxiety.

### Findings

Three means by which humor buffers against death anxiety, were identified through the memes individuals distributed in light of the COVID-19 pandemic outbreak: a) Humor as a means for connecting to cultural worldviews b) Humor as a means for inclusion in a group c) Humor as a means to gain a sense of control. All three means relate to the buffer mechanisms mentioned above.

### Humor as a means for connecting to cultural worldviews

Culture acts as a death anxiety buffer, as many specific beliefs and behaviors that define cultural worldviews offer symbolic immortality [11]. At times such as during a life-threatening pandemic outbreak, cultural worldviews, beliefs, and behaviors become ambiguous thus threatening individuals’ ability to buffer against death anxiety. In this regard, research has shown that tolerance for ambiguity moderates the effect of mortality salience on death anxiety [46, 47]. Therefore, humor may facilitate tolerance to ambiguity as it often addresses the apparent discordance between reality and one’s personally held cultural worldview [22]. Thus, discordance, unsuitability, or the violation of expectations that are common components in humor [48] may be relevant to its potential use as an existential anxiety buffer.

In Israel, great cultural importance is given to children and childbearing. This centrality of children has been explained by the importance of procreation in the Jewish religion, as well as perceived threats to the survival of the Jewish people in general, and the State of Israel in particular (e.g., [49–51]). Thus, during the COVID-19 outbreak when the reality of parents being in quarantine with their children and emotionally stressed over that situation, contradicts cultural worldviews that emphasize the joy in children and childbearing, there is a need to use humor in that context as indeed found in many memes.

A good example is a meme concerning the social imperative of wanting to have and raise children: *"Corona death rate*: *2*.*4% of patients*, *quarantine with children death rate*: *100% of parents"; " My wife and I decided we don’t want to have children*. *We’ll let them know tonight…"*. Or memes contradicting the cultural view on children as a source of joy or even life itself: *"I’m not crying*, *it’s just that summer vacation got stuck in my eye in the middle of March"; "There are also some benefits in unlimited time at home with two small children*. *I*, *for example*, *am no longer afraid of death"*.

Another cultural worldview relevant to Israeli society is marriage. Israel is characterized by high marriage rates, low divorce rates, and a younger median age at first marriage compared to most of the Western world [52, 53]. Nevertheless, the reality of being in quarantine together may contradict couples’ socially endorsed values, as reflected in the following memes: *"First case of confirmed Coronavirus death in Israel*. *The subject is a married man strangled by his wife after four days in quarantine"; "Today at 8*:*00 pm*, *all divorced and single people go out on the porch and applaud all the married people in quarantine"; "For only 3500$ we come to your house dressed as a coronavirus rescue team to rescue you from your wife*. *we take you to your girlfriends’ place for 14 days of quarantine and bring you back home afterward"*.

Culture also provides a buffer against anxiety by providing a set of values and normative standards against which an individual may be judged a worthwhile, socially acceptable person. The goal is to feel that one is a valuable member of a meaningful culture, which in turn evokes a feeling of symbolic immortality that mitigates the fear of finitude [54]. Visual appearance has great social and cultural significance to individuals in modern–western culture and has a marked effect on individuals’ social, cultural, and economic status [55]. Scholars of fashion note that fitting in with one’s social group and with society at large requires adopting dress and appearance that correspond to prevailing social norms [56]. In quarantine, keeping a socially endorsed appearance, hence social norms and values is hard. Thus, memes relate to the contradiction between quarantine reality and social behaviors regarding physical appearance: *"My fluid diet is working great*. *After 4 glasses of wine*, *I don’t care that I’m fat"; Bibi [Israel’s prime minister]*, *release the beauticians from quarantine*, *this is the second time this week that I’ve called my female neighbor—bro"*.

Another way of buffering against the mortality salience evoked by the life-threatening pandemic outbreak is to feel that one is a valuable member of meaningful culture, by maintaining a sense of group membership. This idea will be addressed in the next theme.

### "Mad World": Humor as a means for inclusion in group

As mentioned above, social distancing and quarantine along with death are members of a broad category of “emergency situations that can be remedied through coalitional support” (p. 373) [21]. Thus, individuals primed with the threat to their health and with the threat of being isolated from important social relationships would respond in similar ways to those in previous TMT studies who were primed with death [19] i.e., motivated toward inclusion in groups. When mortality is salient due to a life-threatening pandemic and social distancing, humor can be used for maintaining a sense of group membership [31]: *"We’ll survive the Corona*, *I’m not worried*. *But think of our great-great-grandchildren*, *who once a year will have to live in quarantine for two weeks and eat only canned food while singing hymns on toilet paper as part of the Coronavirus holiday customs that come to mark the miracle that God has made for the Israel"; “Your ancestors fought the Nazis and the Arab armies*, *paved roads*, *dried up swamps*. *You are only asked to wash your hands*, *put your ass on the couch and watch Netflix*. *Can you please not screw it up*?*”*.

Memes may also reflect on the fear of being isolated and out of the group such as in the following meme: *"Can anyone update what’s happening with the Coronavirus*? *It has been three minutes since the last update and I suspect everyone died except me"*. And it may reflect on the self as a part of the group going mad such as in the following meme: *"On Sunday we all go out on the balcony and applaud ourselves for not having jumped off yet"*. Both memes relate to death but in a reverse sense than what is normally used. If everyone is dead one wishes to die along with everyone, as long as not to stay alone and out of the group.

Some memes indicate the changed worlds’ rules and norms: *"Once when a person sneezed*, *it was of custom to say ’bless you’*. *Today when a person sneezes*, he is been told F**** the H*** away from here, *go die at home*!*"; "How the world has changed*, *today dogs take their owners*, *mouth gagged*, *for a walk outside"; "20 years ago we had Johnny Cash*, *Bob Hope*, *and Steve Jobs*. *Now we have no cash no hope and no jobs"*. Nevertheless, although the group rules changed and the world has gone mad, one is still a part of this crazy world: *"Applause for doctors and nurses in the psychiatric hospital who are going to absorb thousands of Israelis soon"; "Today when I went out to dump the garbage I saw the neighbor talking to a cat*. *I think she went mad as it is clear that the cat doesn’t understand her*… *I came home*, *told my dog about it*, *and we both laughed"*.

When mortality is salient due to a life-threatening pandemic and social distancing, humor can be also used to maintain boundaries between in-groups and out-groups [31], as reflected in the following meme: *"What do these Chinese people care for putting us all in quarantine*?! *They are not like us; they are stuck at home with only one child*!*"*. This meme may also indicate the aggressions the COVID-19 pandemic outbreak may provoke due to its’ potential threat to human lives as well as perceived lack of control over its circumstances. The way humor may be used to buffer against the lack of control over death’s circumstances, we will address this in the next theme.

### "Gallows humor" as a means of gaining a sense of control

As mentioned above, TMT postulates that humans’ awareness of the inevitability of death, as well as our perceived lack of control over its circumstances, may create death anxiety [11]. To buffer against the death anxiety caused by the lack of control over the circumstances of the life-threatening COVID-19 pandemic, individuals often used gallows humor. Gallows humor is a form of dark humor that makes light of existential concerns, often used in times of predicament as a means to regain control in uncontrollable circumstances [57]. Indeed, some memes contain gallows humor such as the following: *"Test results*: *Cannabis*: *Positive*, *AIDS*: *Positive*, *Cocaine*: *Positive*, *Corona*: *Negative*. *I got stressed out for a minute but everything is fine"*.

Another form of gallows humor refers to previous adversities the Jewish people went through in history such as the persecution of the Jewish People in Europe: *"Let’s not go round and round*, *bottom line*, *when does the pogrom [bloodbath] begin*?*"*; or such as the persecution of the Jewish People by the Nazis: *"Does anyone know when the Nazis come*?*"* These gallows humor memes not only reassert control in uncontrollable circumstances but also reflect on motivation towards endorsement of culture and tradition thus, inclusion in the large cultural Jewish group.

Notably, cultural marks that were chosen for the mentioned above gallows humor memes concern violence and aggression being implemented on the individual, thus may reflect on the terror individuals experience these days. Hence, individuals seek out humor to help them deal with the impact of the stressors, as was evident in a previous study that explored humor as a means that helps victims deal with the psychological damage caused by aggression [58]. Individuals using this kind of humor resent being a victim and use humor to restore a sense of control and power.

As individuals feel under attack by the COVID-19 outbreak, they may also feel tension and aggression. According to the relief theory, humor is the release of pent-up tension or mental energy [59]. This kind of use of humor is reflected in the following memes: *"Truth is that the "Big Brother" [a reality show] format is much nicer now when voting who to send to his death"; "I’m at home with four children all morning*. *He calls at six p*.*m*. *on his way home back from work*: *Honey*, *can I get something on the way*? *Yes*, *I told him*, *stop on the way to buy me a meter and a half rope for tomorrow…"; "Does anyone know what the suicide rates among parents during quarantine with segmentation for the number of children is*?*"; "Wait*, *when they said on the news*, *they are closing schools and kindergartens they meant with the kids inside*? *Right*?*"*

Feelings of hostility, aggression, or sexuality that are expressed bypassing any societal norms are said to be enjoyed [59]. This brings us back to the first theme indicating how discordance, incongruity, or the violation of expectations that are common ingredients in humor [48] may be relevant to its potential use as an existential anxiety buffer.

## Discussion

Our findings indicate three means of using humor to buffer against death anxiety, individuals experience in light of the COVID-19 pandemic outbreak. Exploration of memes indicated that humor was used as a means for connecting to cultural worldviews, as a means for inclusion in a group, and as a means to gain a sense of control. These means of buffering against death anxiety are in line with previous studies, indicating different yet similarly intended coping strategies with death anxiety during the COVID-19 pandemic. For instance, research shows how the state and forms of nationalism have been strengthened worldwide hence in many countries, self-isolated residents have been waving flags and singing national anthems [60, 61]. Research also shows that social connection, achievement, and religion all played an important role in warding off death anxiety caused by the pandemic [62]. Although these are all important means of buffering against death anxiety, our findings offer another important dimension of coping with death anxiety caused by the COVID-19 pandemic following De Wall and Baumeister’s reason that: “Clutching happy thoughts may serve the function (central to terror management theory) of preventing the conscious mind from being paralyzed by the terror of death” (p. 984) [63].

TMT postulates that humankind has created several coping mechanisms which aim at warding off death-related cognitions and thoughts and enable everyday functioning [11]. Three common buffers that minimize the anxiety of mortality salience were revealed. The first theme, referring to the affirmation of one’s cultural worldview, indicates how at times when the reality contradicts one’s personally held cultural worldview, thus worldviews and or the reality become obscure, humor may facilitate tolerance to that obscurity to be used as a mean to affirm cultural worldviews. In addition, as reflected in the second theme, which refers to the affirmation of the self and one’s personal views, findings indicate how humor may be used to affirm one is a valuable member of a meaningful culture through maintaining a sense of group membership. Excessive death anxieties show an individual’s powerlessness [64], the belief that one is meeting or exceeding cultural standards and values promotes a sense of power and self-esteem, which is another death anxiety buffer mechanism offered by TMT [65]. Affirmation and endorsement of culture and tradition thus, inclusion in the large cultural Jewish group is also evident in the third theme, namely affirmation of one’s significance in the context of close personal relationships, through the use of gallows humor. Lastly, findings indicate how humor is used to buffer against death anxiety through the release of pent-up tension or mental energy as reflected in the third theme. These findings are consistent with previous research indicating that individuals with a sense of humor tend to be able to stay in and fight any less conducive situations [66] and that humor can function as an effective means of coping with stressful or traumatic experiences [67, 68]. Moreover, in research conducted by Boerner et al., a sense of humor was negatively associated with emotion regulation difficulties and negative changes and it was useful to cope with trauma [69].

### Limitations and implications

This research was restricted to 237 memes distributed in Israel and collected by the two authors, both of which are females in their 40^th^ and who hold a Ph.D. This is a narrowly defined sample, and it might be that individuals of different ages, different backgrounds, and different gender may come across a different use of humor as maybe they have less/ more time and technology availability to engage in receiving and sharing memes. Future research should reveal how humor may act as a way to buffer against death anxiety among a larger, more heterogeneous sample of recipients. Furthermore, future research should focus on memes and the age of people engaging with them as there might be generation gaps in meme humor and how it is shared. In addition, future research may benefit from the inclusion of a comparative dimension with cultural material from other countries to distinct specific culturally endorsed means of using humor as a buffer against death anxiety. Moreover, future research may use interviews with individuals distributing humorous memes to explore the way they perceive humor as a means to buffer against death anxiety. Lastly, quantitative studies should also explore the way individuals use humor as a strategy to buffer against death anxiety. For example, the quantitative inquiry may be helpful to gain knowledge about the diffusion and ‘usage or interaction’ with the content of mems.

Despite these limitations, our research supports previous research indicating humor as a means to buffer against death anxiety [22, 70], thereby allowing individuals to reappraise their position as being over and above any challenge; taking back power and control over the given situation [71]. The current study supports previous research and explores how humor may act as a means to buffer against death anxiety according to TMT. Thus, our research yields important implications for practice. As the current research findings suggest that coping humor can buffer against death anxiety, we encourage health care professionals to use humor as well as to teach clients to use humor as a useful coping strategy. Notably, some hospitals in Israel and the USA have formal humor programs in which staff provide laughter rooms, therapeutic clowns, and comedy carts filled with humorous books, videos, and other items for patients [23]. Therefore, our research indicates the death-anxiety-buffering potential that lies in such methods, especially in stressful times of a global health pandemic.
